# Anti-angiogenic therapy for advanced primary pulmonary lymphoepithelioma-like carcinoma: a retrospective multicenter study

**DOI:** 10.1007/s00432-022-03935-0

**Published:** 2022-04-04

**Authors:** Hejing Bao, Ling Zhen Ma, Chengzhu Zhao, Mengge Yu, Baishen Zhang, Juan Zhang, Guibao Peng, Xiaotong Lin, Yinhua Fang, Hehong Bao, Shudong Ma

**Affiliations:** 1 Department of Oncology, Nanfang Hospital, Southern Medical University, Guangzhou, 510515 Guangdong China; 2 Department of Oncology, Chongqing University Three Gorges Hospital/Chongqing Three Gorges Central Hospital, Chongqing, 404100 China; 3 Department of Emergency, Chongqing Banan District People’s Hospital, Chongqing, 401320 China; 4 Department of Oncology, Sun Yat-Sen University Cancer Centre, State Key Laboratory of Oncology in South China, Guangzhou, Guangdong China; 5 Department of Otorhinolaryngology, Zhujiang Hospital, Southern Medical University, 253 Gongye Road, Guangzhou, 510282 Guangdong China; 6 Department of General Surgery, The Fifth Affiliated Hospital of Guangzhou Medical University, Guangzhou, China; 7 Oncology Center, The First Affiliated Hospital of Guangzhou University of Chinese Medicine, Guangzhou, China

**Keywords:** PPLELC, Anti-angiogenic therapy, Efficacy and safety, Retrospective study

## Abstract

**Purpose:**

Primary pulmonary lympho-epithelioma-like carcinoma (PPLELC) is a rare subtype of primary non-small cell lung cancer (NSCLC). Currently, there is still lack of research data on anti-angiogenic therapy of advanced PPLELC. The purpose of this study was to investigate the efficacy and safety of anti-angiogenic therapy combined with chemotherapy compared with traditional chemotherapy for these patients.

**Methods:**

Advanced PPLELC patients admitted to six grade A hospitals from January 2013 to January 2021 were selected. The patients received anti-angiogenic therapy combined with chemotherapy (AT group) or chemotherapy (CT group) alone.

**Results:**

A total of 65 patients were included in this study, including 31 patients in the AT group treated with anti-angiogenic therapy combined with chemotherapy and 34 patients in the CT group treated with chemotherapy alone. As of October 1, 2021, the median progression-free survival (PFS) in the AT group was 11.2 months [95% confidence interval (CI), 5.9–16.5]. The median PFS in the CT group was 7.0 months [95%CI, 5.1–8.9] [Hazard Ratio (HR), 0.49; 95%CI, 0.29–0.83; *P* = 0.008]. The 1-year PFS rates were 41.9% and 17.6%, respectively. The overall response rates (ORR) of two groups were 45.2% (95% CI, 0.27–0.64), 38.2% (95% CI, 0.21–0.56), (*P* = 0.571). The disease control rates (DCR) of two groups were 93.5% (95% CI, 0.84–1.03), 88.2% (95% CI, 0.77–1.00), (*P* = 0.756).

**Conclusion:**

Among patients with advanced PPLELC, the PFS of patients with anti-angiogenic therapy combined with chemotherapy is better than that of patients with chemotherapy alone. Anti-angiogenic therapy combined with chemotherapy is an optional treatment scheme.

**Supplementary Information:**

The online version contains supplementary material available at 10.1007/s00432-022-03935-0.

## Introduction

Lympho-epitheliomatoid carcinoma is a rare epithelial neoplasm originating mostly in the nasopharynx and also in the foregut (Ambrosio et al. 2011; Anand et al. 2018). Primary pulmonary lympho-epithelioma-like carcinoma is a rare subtype of primary non-small cell lung cancer that histologically resembles undifferentiated nasopharyngeal carcinoma (NPC) (Hayashi et al. 2012), the incidence in all cases of non-small cell lung cancer is about 0.7% (Anand et al. 2018; Xie et al. 2020). It was previously classified as a variant of large cell carcinoma (Travis et al. 2006), then reclassified as other and unclassified cancers in the 2015 World Health Organization (WHO) Classification of Tumors of the Lung (Travis et al. 2015).

First described by Bégin et al. (1987), PPLELC has been considered to be closely associated with Epstein–Barr virus (EBV) infection (Han et al. 2000). About 1600 cases have been reported worldwide in the past 33 years since the discovery (Kim et al. 2016; Darrason et al. 2019; Hong et al. 2019; Wu et al. 2020; Chen et al. 2021), mainly focused on the past five years, reporting mainly in Asia, especially Hong Kong, Taiwan, Guangdong and other regions. PPLELC usually affects never-smokers, is gender-neutral, and is younger than non-small cell lung cancer (Lin et al. 2016). Similar to other types of lung cancer, the prognosis is good for most patients diagnosed early with lung lympho-epithelioid, with a median overall survival of approximately 107 months and a 5-year survival of approximately 60%, compared with a mean survival of 13 months for patients with non-lung lympho-epithelioid (He et al. 2015; Liang et al. 2012).

The main treatment strategy for early PPLELC is surgery (Lin et al. 2016; Ho et al. 2006). For patients with distant metastasis, surgical treatment is not possible, and a variety of treatment modes are usually accepted, including chemotherapy, radiotherapy and immunotherapy (Kumar et al. 2017; Narayanan et al. 2019; Zhou et al. 2019; Tang et al. 2020; Qiu et al. 2019). Several treatments have been reported in the literature, but the treatment of PPLELC is empirical due to its rarity. However, there is still a lack of research data on anti-angiogenic therapy in advanced PPLELC. Therefore, this study aims to investigate the effect of anti-angiogenic therapy on advanced PPLELC patients.

## Materials and methods

### Patients

**Fig. 1:**
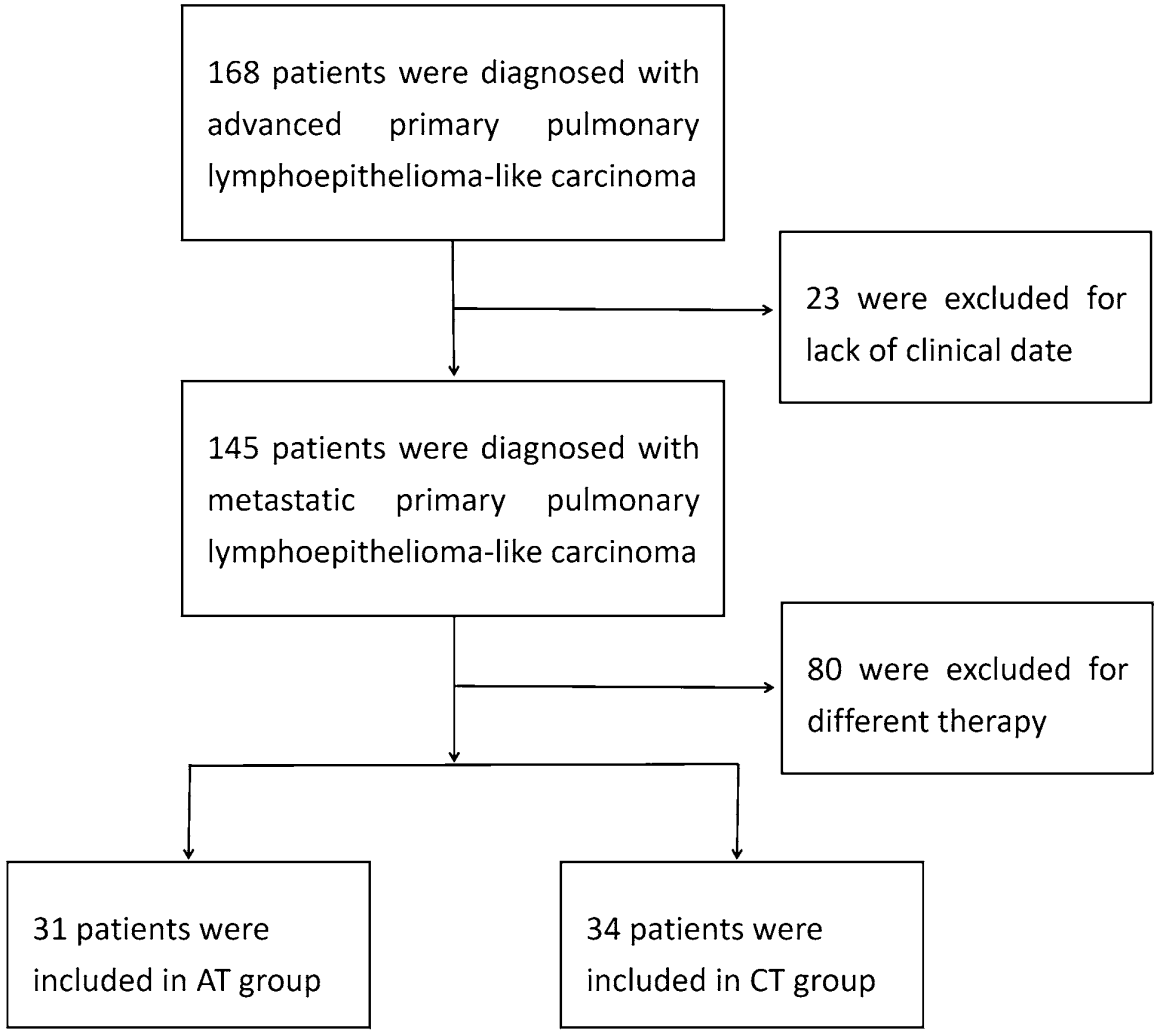
Study enrollment. A total of 168 patients diagnosed with advanced PPLELC in six grade A hospitals from January 2013 to January 2021 were selected, and 65 patients were eventually included. Due to the lack of clinical data, 23 patients were excluded. 80 patients were excluded because the treatment regimen did not meet the requirements of the study. Finally, of the 65 included patients, 31 patients were included in the AT group combined with first-line anti-angiogenesis therapy, and 34 patients were included in the CT group with first-line chemotherapy

Patients aged 18 years or older, stage IIIB-IV, diagnosed as lung lympho-epithelioid carcinoma according to the 2015 WHO histological classification criteria for lung tumors, and without allergenic EGFR or ALK mutations; According to the Solid Tumor Response Assessment Criteria (RECIST), version 1.1 has at least one measurable lesion. This retrospective study collected data of PPLELC patients from Nanfang Hospital affiliated to Southern Medical University, Zhujiang Hospital affiliated to Southern Medical University, The First Affiliated Hospital of Guangzhou University of Traditional Chinese Medicine, Cancer Hospital of Sun Yat-sen University, Cancer Hospital affiliated to Guangzhou Medical University, Three Gorges Hospital affiliated to Chongqing University from January 2013 to January 2021. 65 patients with advanced PPLELC were eventually included in the study (Fig. 1).

### Experimental design and treatment

In this real-world retrospective study, patients were divided into AT group treated with anti-angiogenic therapy combined with chemotherapy and CT group treated with chemotherapy. AT group received anti-angiogenic therapy, including bevacizumab (*n* = 22), endu (*n* = 6) or anlotinib (*n* = 3), the chemotherapy regimen included paclitaxel (*n* = 14), pemetrexed (*n* = 10) or gemcitabine (*n* = 7) combined with platinum or mono-therapy. The chemotherapy regimen in CT group included paclitaxel (*n* = 19), pemetrexed (*n* = 10) or gemcitabine (*n* = 5) combined with platinum or mono-therapy.

### Assessment

The tumor response assessment program is conducted every two to four cycles. Tumor response was assessed as complete response (CR), partial response (PR), disease stability (SD), or disease progression (PD) according to RECIST version 1.1. Adverse events and laboratory abnormalities were graded according to the National Cancer Institute Standard for Common Terminology for Adverse Events, Version 4.0.

### Statistical analysis

Efficacy was assessed in the enrolled population and safety was assessed in the treated population. Kaplan–Meier methods were used to estimate progression-free survival. *T* test was used for measurement data (age) and Chi-square test (Cartesian test) was used for counting data. Univariate Cox regression was used to screen individual risk factors. The independent prognostic covariate of PPLELC patients was determined by multivariate Cox regression model and forward procedure. Cox proportional risk regression was used for univariate and multivariate survival analyses. All statistical analyses were performed in IBM SPSS Statistics (Version 19.0, Armonk, NY, USA). When *P* value was less than 0.05, the difference was considered statistically significant.

## Results

### Patient characteristics

The median age of patients was 54 years (interquartile range, 48–62 years). There were 38 female patients (58.5%) and 27 male patients (41.5%). ECOG score was 0 in 24 patients (36.9%) and 1 in 41 patients (63.1%). 18 patients (27.7%) were smokers, and 10 patients (15.4%) had a family history. There were 10 patients (15.4%) in stage IIIB and 55 patients (84.6%) in stage IV. Patients with single organ metastasis accounted for 19 (29.2%), and patients with multiple organ metastasis accounted for 46 (70.8%). The most common tumor sites were middle lobe of right lung and lower lobe of right lung, with 18 (27.7%) and 17 (26.1%) patients, respectively. There were 61 patients (95.3%) who were positive for Epstein–Barr virus, 15 patients (23.1%) had received radiotherapy, and 4 patients (6.2%) with gene mutations, including 3 gene mutations in the AT group, which were NRAS Q61L (14.5%) missense mutation, SMAD4 K110Ter (19.5%) mutation and FGFR3–TACC3 (F17:T10) fusion. MYC amplification was found in 1 case in CT group. There was no significant difference between the two groups at baseline (*P* > 0.05). (Table1).

**Table 1:** Baseline characteristics

	All patients (*n* = 65)	AT group (*n* = 31)	CT group (*n* = 34)	*P*
Age, years Median(IQR)	54 (48–62)	55 (48.5–61.5)	54 (48–61.25)	0.892
Sex				0.285
Female	27 (41.5%)	15 (48.4%)	12 (35.3%)	
Male	38 (58.5%)	16 (51.6%)	22 (64.7%)	
ECOG performance status				0.592
0	24 (36.9%)	12 (38.7%)	11 (32.3%)	
1	41 (63.1%)	19 (61.3%)	23 (67.6%)	
Smokers	18 (27.7%)	10 (32.3%)	8 (23.5%)	0.432
Family history	10 (15.4%)	3 (9.7%)	7 (20.6%)	0.309
TNM				0.382
III B	10 (15.4%)	3 (9.7%)	7 (20.6%)	
IV	55 (84.6%)	28 (90.3%)	27 (79.4%)	
Metastases				0.260
Single organ	19 (29.2%)	7 (22.6%)	12 (35.3%)	
Multiple organ	46 (70.8%)	24 (77.4%)	22 (64.7%)	
Tumor location				0.903
Left upper lobe of lung	10 (15.4%)	5 (16.1%)	5 (14.7%)	
Left lower lobe of lung	15 (23.1%)	8 (25.8%)	7 (20.6%)	
Right upper lobe of lung	5 (7.7%)	2 (6.5%)	3 (8.8%)	
Right middle lobe of lung	18 (27.7%)	7 (22.6%)	11 (32.4%)	
Right lower lobe of lung	17 (26.1%)	9 (29.0%)	8 (23.5%)	
EB( +)	61 (95.3%)	29 (93.5%)	32 (94.1%)	1.0
Radiotherapy	15 (23.1%)	6 (19.3%)	9 (26.5%)	0.496
Gene mutation	4 (6.2%)	3 (9.7%)	1 (2.9%)	0.540

Data are median number (IQR) or *n* (%). *IQR* interquartile range. Data from all patients who were enrolled in this study

### Efficacy

The minimum follow-up time was about 1.4 months. Median follow-up time was 18 months in the AT group (95% CI, 13.5–26) and 19 months in the CT group (95% CI, 13–28). AT the end of the follow-up period, 11 patients (35.5%) in the AT group and 12 patients (35.3%) in the CT group continued treatment. Anti-angiogenic therapy was administered either as up-front or salvage therapy (first-line *n* = 18, ≥ second-line *n* = 13, cross-line *n* = 7), while chemotherapy was used in the first-line therapy.

**Fig. 2:**
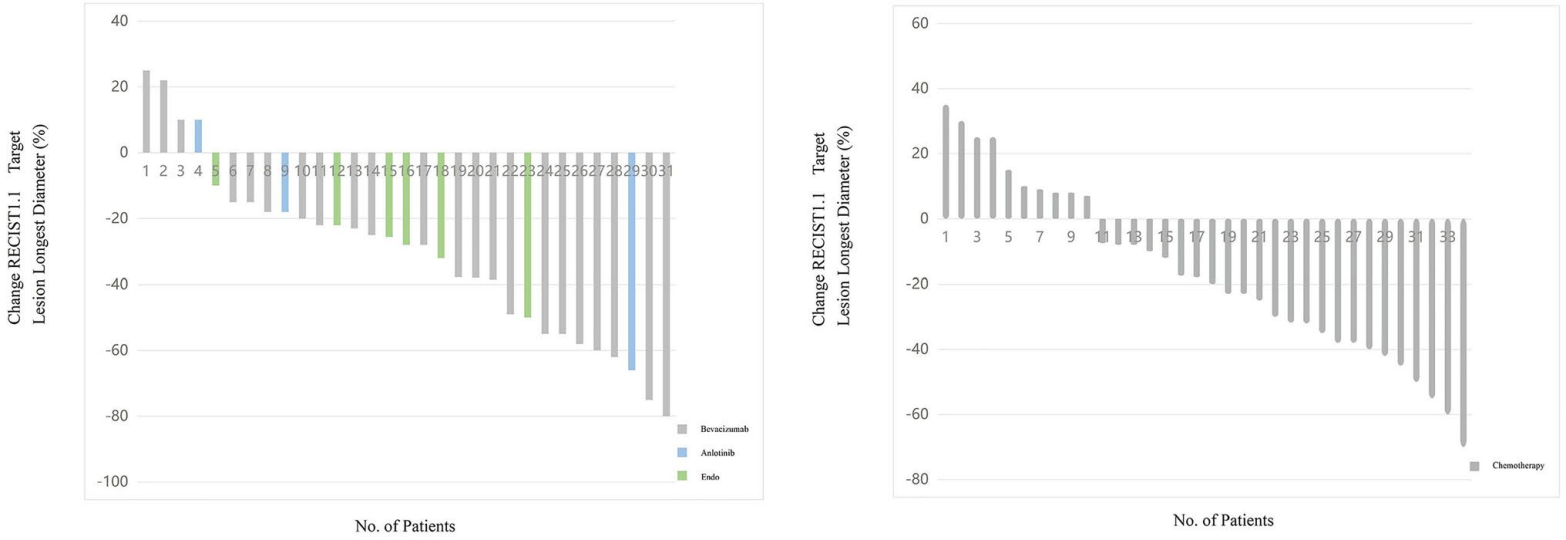
Waterfall diagram of optimal efficacy evaluation for patients in the AT group and CT group. **A** In the AT group, 15 patients achieved SD status, 14 patients achieved PR status, and 2 patients did not respond to treatment. **B** In the CT group, 17 patients achieved SD status, 13 patients achieved PR status, and 4 patients did not respond to treatment

There were no CR cases in both AT group and CT group. In AT group, 14 patients (45.2%) reached PR, 15 patients (48.3%) reached SD, and 2 patients (6.5%) did not respond to treatment. In CT group, 13 patients (38.2%) reached PR, 17 patients (50.0%) reached SD, and 4 patients (11.8%) did not respond to treatment. The ORR of AT group and CT group were 14 (45.2%, 0.27–0.64) and 13 (38.2%, 0.21–0.56), *P* = 0.571; DCR were 29 (93.5%, 0.84–1.03) and 30 (88.2%, 0.77–1.00), *P* = 0.756 (Table 2, Fig. 2).

**Table 2:** Efficacy of treatments

	AT group (*n* = 31)	CT group (*n* = 34)	*P*
Best overall response			0.763
Complete response	0	0	
Partial response	14 (45.2%)	13 (38.2%)	
Stable disease	15 (48.3%)	17 (50.0%)	
Progressive disease	2 (6.5%)	4 (11.8%)	
Objective response	14 (45.2%, 0.27–0.64)	13 (38.2%, 0.21–0.56)	0.571
Disease control	29 (93.5%, 0.84–1.03)	30 (88.2%, 0.77–1.00)	0.756

Data are *n* (%). Confirmed complete and partial responses were assessed by the investigator according to the Response Evaluation Criteria in Solid Tumors, version 1.1

**Fig. 3:**
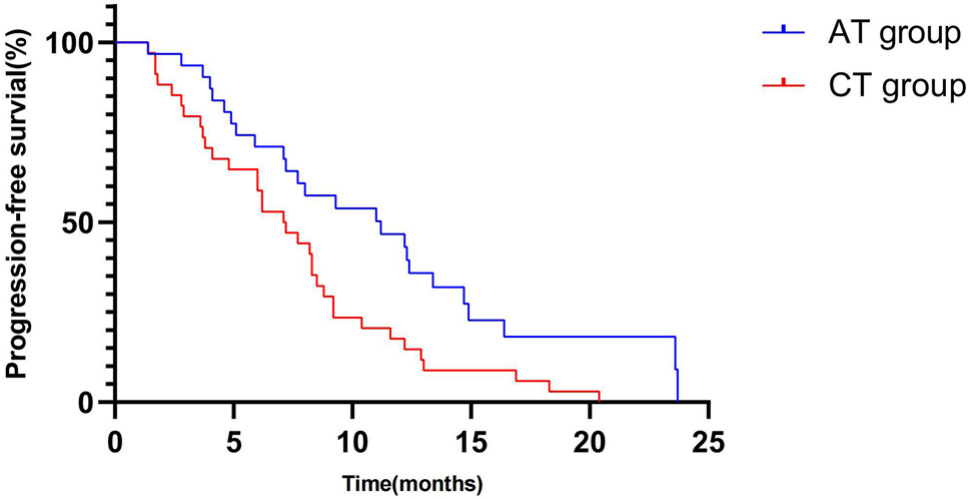
PFS in AT group and CT group. Median PFS was 11.2 months in the AT group [95% CI, 5.9–16.5] and 7.0 months in the CT group [95% CI, 5.1–8.9] [HR, 0.49; 95% CI, 0.29–0.83; *P* = 0.008]

The median PFS was 11.2 months in the AT group [95% CI, 5.9–16.5] and 7.0 months in the CT Group [95% CI, 5.1–8.9] [HR, 0.49; 95% CI, 0.29–0.83; *P* = 0.008]. The 1-year PFS rates were 41.9% and 17.6%, respectively (Fig. 3, Supplement Table 1).

**Fig. 4:**
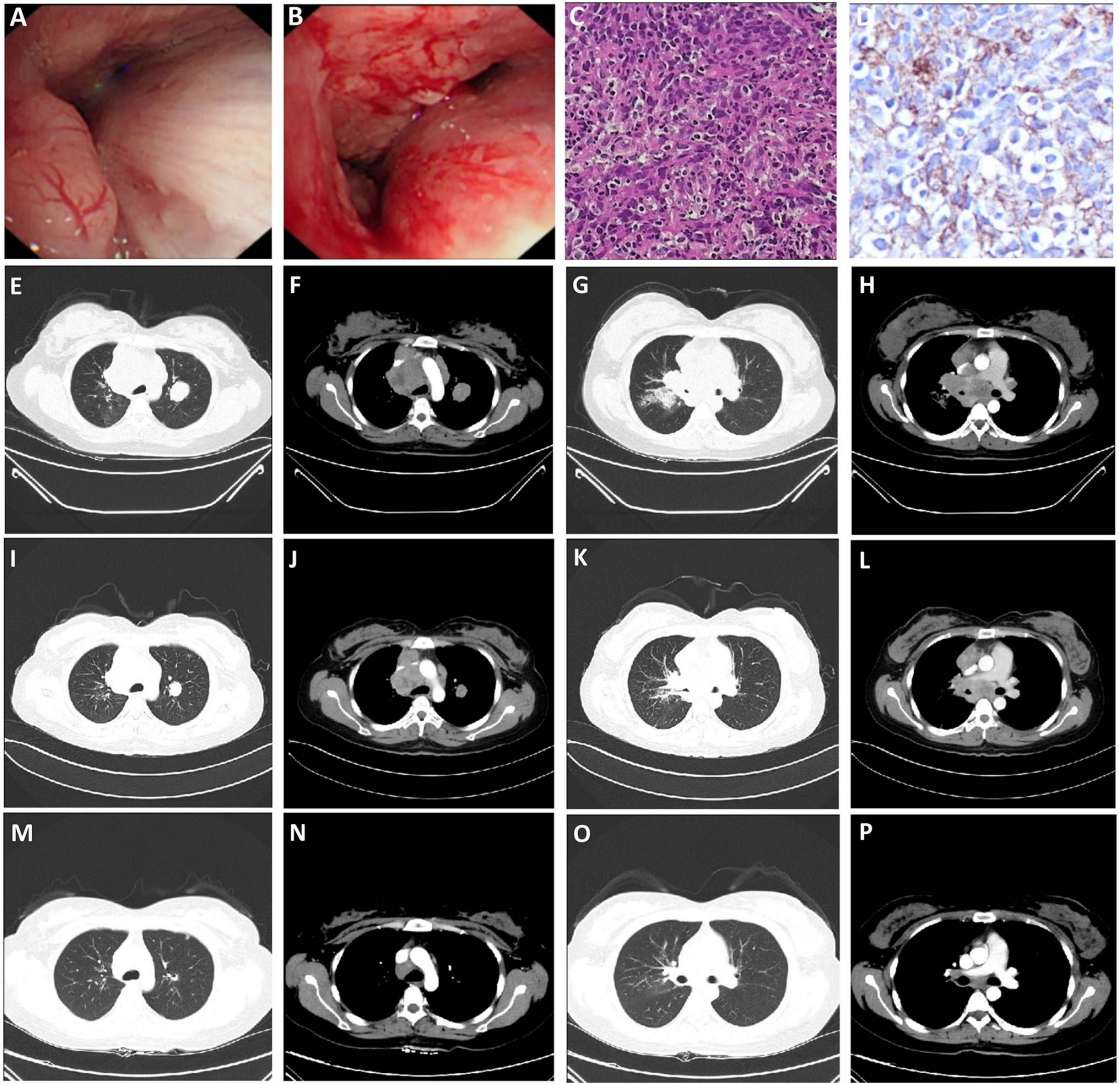
Radiographic images of the patient with FGFR3–TACC3 (F17:T10) fusion. **A**, **B** Fiber-bronchoscopy results in October 2018 indicated the growth of new organisms in the right main bronchus; **C** HE × 400 times, the results showed that some tumor cells were arranged in nests, with abundant cytoplasm, red staining, obvious nuclear atypia, interstitial fibrous hyperplasia accompanied by a large number of lymphocytes. **D** Immuno-histochemical × 400 times: PD-L1(22C3) CPS = 80; **E**–**H** Enhanced chest computed tomography images in October 2018, mass soft tissue density shadow near hilum in the upper lobe of right lung, with obstructive pneumonia, multiple lymph node metastases in mediastinum and bilateral hilum, and double lung metastases (**E** and **G** are pulmonary window, **F** and **H** are mediastinal window); **I**–**L** Enhanced chest computed tomography images in February 2019 showed that the mass soft tissue density shadow near hilum in the upper lobe of right lung was smaller than before, obstructive pneumonia was better than before, multiple lymph node metastases in mediastinum and bilateral hilum were smaller than before, and double lung metastases were smaller than before (**I** and **K** are lung window, **J** and **L** are mediastinal window); **M**–**P** Enhanced chest computed tomography images in June 2019 showed that the mass soft tissue density shadow near hilum in the upper lobe of the right lung was smaller than before, obstructive pneumonia was better than before, multiple lymph node metastases in mediastinum and bilateral hilum were smaller than before, and double lung metastases were smaller and less than before (**M** and **O** are lung window, **N** and **P** are mediastinal window)

In this study, we reported a 59-year-old female patient with FGFR3–TACC3 (F17:T10) fusion, no smoking history, tumor located in the right lung with lymphatic, liver, and bone metastases, clinical stage T4N3M1, pathologically confirmed as PPLELC, Genetic analysis revealed FGFR3–TACC3 (F17:T10) fusion without EGFR, ALK and other conventional mutations. The patient was treated with anlotinib in combination with albumin–paclitaxel and carboplatin at the physician's recommendation, achieving disease control and PFS for 8 months (Fig. 4).

### Safety

Treatment-related adverse events (both hematological and non-hematological poisoning events) occurred more frequently in the AT group than in the CT group. In the AT group, there were 17 cases (54.8%) of grade 3 and 8 cases (25.8%) of grade 4 treatment-related adverse events. There were 13 cases (38.2%) of grade 3 and 12 cases (35.3%) of grade 4 treatment-related adverse reactions in the CT group. The most common adverse reactions in AT group were decreased appetite 7 (22.6%), anemia 8 (25.8%), leukopenia 14 (45.2%), neutropenia 16 (51.6%), and decreased platelet 8 (25.8%). Anemia 10 (29.4%), leukopenia 14 (41.2%), neutropenia 15 (44.1%), and thrombocytopenia 10 (29.4%) were most common in the CT group (Table 3).

**Table 3:** Treatment-related adverse events

	AT group (*n* = 31)				CT group (*n* = 34)			
	Grade 1–2	Grade 3	Grade 4	ALL	Grade1–2	Grade3	Grade4	ALL
Fatigue	4 (12.9%)	0	0	4 (12.9%)	2 (5.9%)	0	0	2 (5.9%)
Nausea	6 (19.3%)	0	0	6 (19.3%)	5 (14.7%)	0	0	5 (14.7%)
Vomiting	4 (12.9%)	0	0	4 (12.9%)	4 (11.8%)	0	0	4 (11.8%)
Anorexia	7 (22.6%)	0	0	7 (22.6%)	6 (17.6%)	0	0	6 (17.6%)
Peripheral sensory neuropathy	2 (6.5%)	0	0	2 (6.5%)	2 (5.9%)	0	0	2 (5.9%)
Hypertension	2 (6.5%)	2 (6.5%)	0	4 (12.9%)	0	0	0	0
Proteinuria	2 (6.5%)	0	0	2 (6.5%)	0	0	0	0
Anemia	8 (25.8%)	0	0	8 (25.8%)	10 (29.4%)	0	0	10 (29.4%)
White blood cell decreased	10 (32.3%)	4 (12.9%)	0	14 (45.2%)	9 (26.4%)	2 (5.9%)	3 (8.8%)	14 (41.2%)
Neutrophil count decreased	2 (6.5%)	6 (19.3%)	8 (25.8%)	16 (51.6%)	3 (8.8%)	3 (8.8%)	9 (26.4%)	15 (44.1%)
Lymphocyte count decreased	4 (12.9%)	2 (6.5%)		6 (19.3%)	3 (8.8%)	3 (8.8%)	0	6 (17.6%)
platelet count decreased	6 (19.3%)	2 (6.5%)	0	8 (25.8%)	6 (17.6%)	4 (11.8%)	0	10 (29.4%)
ALT increased	2 (6.5%)	0	0	2 (6.5%)	0	1 (2.9%)	0	1 (2.9%)
AST increased	2 (6.5%)	0	0	2 (6.5%)	2 (5.9%)	0	0	2 (5.9%)
Blood bilirubin increased	2 (6.5%)	0	0	2 (6.5%)	2 (5.9%)	0	0	2 (5.9%)
Rash maculo-papular	5 (16.1%)	0	0	5 (16.1%)	2 (5.9%)	0	0	2 (5.9%)
Tracheal fistula	0	1 (3.2%)	0	1 (3.2%)	0	0	0	0
Overall	68	17	8	93	56	13	12	81

## Discussion

PPLELC is a rare subtype of NSCLC. In this era of targeted therapy and checkpoint inhibitors for non-small cell lung cancer, anti-angiogenic drugs still play an important role (Shukla et al. 2020). However, the prognosis of advanced PPLELC is poor, due to the lack of drug treatment data, the role of anti-angiogenic therapy in PPLELC is unknown. It is worth exploring the effect of anti-angiogenic therapy in PPLELC.

Induction of angiogenesis is one of the 10 characteristics of malignant tumors. Angiogenesis provides essential nutrients for tumor growth and is an important prerequisite for tumor hematogenous dissemination. The ECOG-4599 study established the status of bevacizumab combined with first-line chemotherapy (Sandler et al. 2007). Small molecule tyrosine kinase inhibitor (TKI) drugs targeting angiogenesis have become one of the research hotspots in recent years. The biggest feature of small molecule drugs is that their target coverage is more comprehensive. In addition to VEGF/R and other pathways, they can also cover PDGF/R, FGF/FGFR and other pathways.

In this study, a patient with FGFR3–TACC3 fusion was reported. In general, operable FGFR3 gene fusion is found relatively commonly in glioma and bladder cancer (Wu et al. 2013), FGFR3–TACC3 fusion has been reported in 2.5% of NPC (Yuan et al. 2014). However, FGFR3 alteration was rarely observed in NSCLC, and was detected in 0.1% of adenocarcinomas and 0.6% of squamous cell carcinomas, respectively (AACR Project GENIE Consortium 2017; Qin et al. 2019). The prevalence of FGFR3 in LELC was 4% (Chau et al. 2065). FGFR3–TACC3 is reported to be a relapsing drug resistance mechanism that can bypass EGFR blockade by all generations of EGFR TKI in NSCLC (Ou et al. 2017). In this study, FGFR3–TACC3 fusion patients were treated with small molecule multi-target TKI, which inhibited VEGFR, PDGFR, FGFR and c-Kit targets, showing anti-tumor angiogenesis and tumor growth inhibition. PFS benefits were obtained in combination with chemotherapy, suggesting that FGFR3 aberrations may represent an opportunity to target therapy in PPLELC.

However, anti-angiogenic therapy alone does not significantly improve patient outcomes, as the removal of blood vessels transforms tumor cells into a hypoxic-tolerant phenotype. Combination of anti-angiogenic therapy with other therapies, including chemotherapy, radiotherapy, immunotherapy and anti-EGFR therapy, has good efficacy due to the vaso-normalization effect induced by anti-angiogenic agents (Tian et al. 2020). Radiation alone as the sole means of treating cancer often triggers the angiogenesis pathway, leading to upregulation of multiple angiogenic growth factors, and anti-angiogenic inhibitors can help overcome this fact (Rani and Prabhu 2021). Randomized Phase III studies have also shown that treatment with antiangiogenic agents in combination with programmed cell death ligand 1 (PD-L1) antibodies significantly improves survival compared with standard therapy in renal cell carcinoma (RCC), NSCLC and hepatocellular carcinoma (HCC) (Hack et al. 2020). However, there are still many problems to be solved, such as how to choose a more reasonable combination partner for anti-angiogenic therapy, the timing, recommended dosage and the proportion of anti-angiogenic inhibitor combination therapy (Tian et al. 2020). In this study, anti-angiogenic therapy combined with chemotherapy in patients with advanced PPLELC was included. The results showed that compared with chemotherapy alone, it can improve the short-term efficacy of patients. It is well known that immunotherapy can improve the long-term prognosis of patients. If anti-angiogenic combined with immunotherapy and chemotherapy is a better choice, it is worth further exploration.

Our study found that anti-angiogenic therapy combined with chemotherapy was more beneficial than chemotherapy alone in patients with advanced PPLELC, but there was no way to screen the dominant population, and some patients did not respond to anti-angiogenic therapy combined with chemotherapy. Both targeted therapy and immunotherapy have corresponding markers to accurately identify the population, but there is no recognized marker in the field of anti-angiogenic therapy. Bevacizumab is a monoclonal antibody with a clear target of VEGF, so it is logical that VEGF expression might predict benefit. However, clinical studies have found that VEGF expression does not predict the benefit of bevacizumab addition (Otrock et al. 2011). The tumor vasculature is a target for antiangiogenic drugs, but quantitative or qualitative measures of the vasculature cannot be used as biomarkers of efficacy because there is no standardized method for measuring micro-vessel density and there is considerable potential for selection and observer bias. The detection of vascular phenotype and tumor cell genotype is still in the stage of preclinical research (Jubb et al. 2006). Compared to using the tissue biomarkers to select effective anti-angiogenic drugs, the studies of discover and validation in the non-invasive, dynamic biomarker were at a more advanced stage, including measuring the circulating protein related to angiogenesis, circulating microRNAs, secrete body outside, circulating endothelial cells and or estimates of progenitor cells and vascular imaging (Paolo et al. 2020).

Our study has some limitations. This study was from a retrospective cohort and the number of included patients was limited. This is due to the rarity of PPLELC and the small number of patients, even fewer patients in the advanced stage of metastasis. Moreover, the emergence and application of different anti-angiogenic drugs in clinical practice will affect the results. Differences in patients' chemotherapy regimens also affected the results. However, this study first reported the value of anti-angiogenic therapy in advanced PPLELC. Data were collected from multiple centers in this study, and patients came from areas with high PPLELC incidence, which has certain advantages.

In conclusion, compared with the current treatment of patients with advanced PPLELC, anti-angiogenic therapy is clinically beneficial and sufficiently safe, and the number of patients should be further expanded or the follow-up period extended to further observe the survival benefit.

## Supplementary Information

Below is the link to the electronic supplementary material.

Supplementary file1 (XLS 30 KB)

## Data Availability

Datasets generated and analyzed during the study are available from HB on reasonable request.
